# Liver Biopsy During Pregnancy: A Review

**DOI:** 10.7759/cureus.98400

**Published:** 2025-12-03

**Authors:** Omar Horani, Idrees Mohiuddin, Zohaib Ahmed, Amna Iqbal, Mohammad W Asif, Mona Hassan

**Affiliations:** 1 Hospital Medicine, The University of Toledo Medical Center, Toledo, USA; 2 Gastroenterology and Hepatology, The University of Toledo Medical Center, Toledo, USA; 3 Neuroscience, The Ohio State University, Columbus, USA

**Keywords:** invasive procedure pregnancy, liver biopsy, liver biopsy pregnancy, liver disease in pregnancy, pregnancy

## Abstract

Liver biopsy remains the gold standard for diagnosing liver pathology, yet its application during pregnancy is limited due to concerns for maternal and fetal safety. While percutaneous, image-guided techniques are generally safe, the risk of complications, though low, has led most providers to defer biopsy until postpartum. Nevertheless, timely evaluation of liver disease in pregnancy is crucial, given its potential impact on both maternal and fetal outcomes. This review examines the indications, outcomes, and safety of liver biopsy during pregnancy.

Pregnancy-related liver disease presents unique diagnostic challenges. Conditions such as acute fatty liver of pregnancy (AFLP), intrahepatic cholestasis of pregnancy (ICP), HELLP (hemolysis, elevated liver enzymes, low platelet count) syndrome, hemophagocytic lymphohistiocytosis (HLH), and hepatic masses may warrant biopsy in select cases. Although diagnostic tools such as the Swansea criteria reduce the need for biopsy in AFLP, histological confirmation may be essential when non-invasive methods are inconclusive. In rare cases of hepatocellular carcinoma or suspected graft rejection post-liver transplant, biopsy may provide indispensable diagnostic clarity. However, evidence regarding its safety remains sparse. Overall, liver biopsy during pregnancy is rarely indicated but can be pivotal when non-invasive tests fail to establish a diagnosis. Current guidelines recommend a stepwise approach, prioritizing non-invasive modalities before proceeding to biopsy. Further prospective studies are needed to better define the safety profile and diagnostic yield of liver biopsy in this population.

## Introduction and background

Liver disease affects approximately 3% of pregnancies worldwide and 0.77% of pregnancies in the United States. Although liver biopsy remains the diagnostic gold standard for parenchymal liver disease, it is infrequently performed during pregnancy due to concerns regarding maternal-fetal safety. In the general population, major biopsy-related complications occur in approximately 1% of cases, with a mortality rate <0.1%. Pregnancy-specific data remain limited, and most available evidence is derived from small cohort studies and case reports.

Liver biopsy is an invasive diagnostic tool that is considered the gold standard to evaluate liver pathology [1]. Percutaneous, image-guided liver biopsy has become the standard method. Although this method is inexpensive, rapid, and quite safe, it is not without risk. Adverse outcomes with this technique are minimal, but not negligible. About 1% of patients undergoing liver biopsy experience serious complications, and the mortality risk is less than 0.1% [2]. Decision-making becomes particularly complex when pregnancy-related liver injury occurs with thrombocytopenia and/or elevated INR (international normalized ratio). Conditions such as HELLP (hemolysis, elevated liver enzymes, low platelet count) and acute fatty liver of pregnancy (AFLP) pose diagnostic and procedural challenges. Liver biopsy remains critical when non-invasive modalities are inconclusive, particularly in overlapping clinical syndromes such as HELLP and AFLP, or in suspected malignancy or graft rejection. Performing liver biopsies during pregnancy is relatively uncommon due to most providers preferring to wait until after birth and to prevent any potential harm to the mother and the fetus [3]. Data on liver biopsy in pregnancy is scarce compared to the data on the general population. Management of liver disease in a pregnant patient is extremely crucial due to the impact on not only the mother, but also the fetus. Through this article, we seek to review some of the indications for liver biopsy during pregnancy and any effects a liver biopsy may have on the fetus and the mother.

## Review

Search strategy

A systematic literature search was performed in PubMed, Embase, Web of Science, and Scopus from January 2000 to February 2025. The following search string was applied and adapted to each database: ("liver biopsy" OR "hepatic biopsy" OR "transjugular liver biopsy" OR "percutaneous liver biopsy") AND ("pregnancy" OR "pregnant" OR "HELLP" OR "acute fatty liver of pregnancy" OR "AFLP" OR "intrahepatic cholestasis of pregnancy" OR "ICP" OR "coagulopathy" OR "thrombocytopenia" OR "graft dysfunction" OR "liver transplant pregnancy"). Manual searches of references from included articles and relevant society guidelines (American Association for the Study of Liver Diseases (AASLD) 2022 [4], European Association for the Study of the Liver (EASL) 2023 [5], British Society of Gastroenterology (BSG) 2024) [6] were also performed to ensure completeness.

Methods

A structured literature review was conducted following a PRISMA-informed framework. A comprehensive search of PubMed, Embase, Web of Science, and Scopus was performed from January 2000 to February 2025 using combinations of the following keywords: “liver biopsy”, “hepatic biopsy”, “transjugular liver biopsy”, “percutaneous liver biopsy”, “pregnancy”, “pregnant”, “HELLP”, “acute fatty liver of pregnancy”, “AFLP”, “intrahepatic cholestasis of pregnancy”, “ICP”, “coagulopathy”, “thrombocytopenia”, and “liver transplant pregnancy”. Reference lists of eligible publications and major society guidelines (AASLD 2022, EASL 2023, BSG 2024) were manually reviewed to identify additional relevant studies. Two independent reviewers screened titles and abstracts, followed by a full-text review of selected articles, with conflicts resolved by consensus. Studies were included if they (1) involved pregnant patients, (2) reported liver biopsy indications, procedural details, or maternal-fetal outcomes, and (3) included laboratory or clinical parameters relevant to biopsy risk assessment. Studies were excluded if they involved non-pregnant cohorts, animal models, in vitro studies, non-hepatic biopsies, conference abstracts without full text, duplicate cohorts, or reports lacking procedural or clinical outcome data. Extracted variables included study design, gestational age at biopsy, indication, biopsy modality (transjugular vs. percutaneous), platelet count and INR at the time of biopsy, anesthesia approach, maternal complications (bleeding, ICU admission, transfusion, mortality), and fetal outcomes (preterm birth, fetal distress, stillbirth, small for gestational age, or SGA). Given substantial heterogeneity in methodology, patient populations, and reported outcomes, a systematic review and a meta-analysis were not performed. Instead, findings were qualitatively synthesized and presented descriptively, with evidence summarized in structured tables detailing biopsy indications, safety thresholds, procedural considerations, and maternal-fetal outcomes.

Indications for liver biopsy during pregnancy

**Figure 1:**
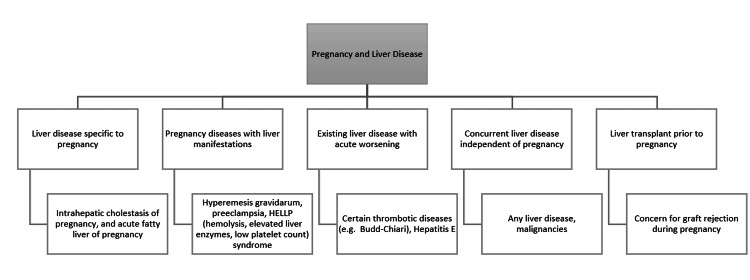
Liver disease and pregnancy Credits: Author's creation

Liver diseases that only occur in pregnancy are seen in 3% of all pregnancies worldwide, and 0.77% of all pregnancies in the United States [7,8]. There are a number of liver conditions that can occur due to pregnancy, including acute fatty liver of pregnancy, acute hepatitis due to a variety of causes (viral, medication-related, and autoimmune), hemophagocytic lymphohistiocytosis (HLH), and intrahepatic cholestasis of pregnancy (ICP). The liver disease during pregnancy can be categorized into five major classes: (1) liver disease specific to pregnancy, (2) pregnancy disease with liver manifestations, (3) existing liver disease with acute worsening, (4) concurrent liver disease independent of pregnancy, and (5) liver transplant prior to pregnancy (Figure 1) [3].

The American College of Gastroenterology (ACG) recommends that the workup for pregnant women with abnormal liver enzymes should be similar to the standard workup as with any non-pregnant patient [9]. The AASLD does not lay out any specific guidelines/recommendations for the workup and management of pregnant female patients with abnormal liver enzymes and/or liver biopsy. In the following sections, we will discuss and summarize the conditions associated with “liver disease and pregnancy” and potential outcomes with respect to liver biopsy in pregnant women. Current guideline consensus (AASLD 2022) supports transjugular liver biopsy (TJLB) as the safest biopsy modality at platelet count ≥50 × 10⁹/L or INR up to 2.0, whereas percutaneous biopsy requires platelets >70-80 × 10⁹/L and INR <1.5 [10].

Acute Fatty Liver of Pregnancy

AFLP is a mitochondrial disease where the fetus is homozygous for long-chain 3-hydroxyacyl-coenzyme. It is due to a dehydrogenase deficiency, which is an enzyme involved in the beta-oxidation of fatty acids. The mother will have intermediate metabolites of fatty acid metabolism in her circulation [11,12]. Laboratory values can include aminotransferase levels from normal to >1000 IU with a bilirubin level below 5 mg/dL upon presentation. Symptoms can include nausea, vomiting, and jaundice, with hepatic encephalopathy and coma in severe cases [13,14]. AFLP is a potentially fatal condition and an obstetric emergency that occurs in the last trimester of pregnancy with an incidence of up to 1/1000 births [15].

Axe and Ch'ng conducted a retrospective review of 20 patients with suspected AFLP between 1993 and 2011; 50% underwent liver biopsies, but all biopsies were postpartum [16]. The Swansea diagnostic criteria are an important tool combining lab values, symptoms, and liver biopsy findings to predict AFLP [7]. Goel et al. evaluated the Swansea criteria in biopsy-proven AFLP in pregnant women and found the following diagnostic parameters: 100% sensitivity, 57% specificity, 85% positive predictive value, and 100% negative predictive value [17]. The Swansea score is calculated on the basis of findings of six or more features in the absence of any other explanation (Table 1) [18]. The caveat of using the diagnostic criteria is that some parameters are only associated with an advanced condition (such as the presence of ascites, encephalopathy, etc.) leading to a challenge with treatment. Zhong et al. noted that none of their 41 AFLP cases would fulfil the Swansea criteria if laboratory testing were taken out of the equation. The authors noted that of six non-laboratory terms in the Swansea criteria, 19.5% of patients fulfilled five terms, 75.6% met four, and 4.9% met three terms before laboratory testing. Furthermore, a modified diagnostic criterion, including gastrointestinal symptoms, abnormal aminotransferases, abnormal bile acid, and coagulopathy, was proposed, yielding a sensitivity and specificity of 97.6% and 96.5%, respectively [18].

**Table 1:** Swansea criteria for diagnosing acute fatty liver of pregnancy Source: Reference [19] Credits: Author's creation

Swansea criteria (require six or more of the following):
· Vomiting
· Abdominal pain
· Polydipsia/polyuria
· Encephalopathy
· Bilirubin > 0.8 mg/dl (14 kmol/l)
· Hypoglycemia
· Uric acid > 5.7 mg/dl (340 kmol/l)
· Leukocytosis > 11,000/mm^3^
· Ascites or bright liver on sonogram
· Aspartate aminotransferase and alanine aminotransferase > 42 IU/l
· Ammonia > 27.5 mg/dl (47 kmol/l)
· Creatinine > 1.7 mg/dl (150 kmol/l)
· Coagulopathy (prothrombin time > 14 s or activated partial thromboplastin time > 34 s)
· Microvesicular steatosis on liver biopsy

Although the Swansea score and lab results can predict AFLP, there are still cases of AFLP that need a liver biopsy to confirm the diagnosis. Onwuagbu et al. described a case of a 35-year-old woman with two previous miscarriages who had a liver biopsy performed three days prior to delivery. The initial differential diagnosis was HELLP syndrome based on the presence of hemolysis, elevated liver enzymes, and low platelets. Seventeen days after her admission, a liver biopsy was performed and AFLP was confirmed on histopathological evaluation. In this report, Onwuagbu makes no mention of the Swansea score, likely due to the patient presentation not associated with typical AFLP. Biopsy proved to be a necessary tool to make the correct diagnosis in this case [20].

HELLP Syndrome

Preeclampsia is a disorder seen after the 20th week of gestation and is characterized by the new onset hypertension and proteinuria. In its severe form, preeclampsia can lead to HELLP syndrome and hepatic compromise (infarction or hematoma) that can lead to high maternal morbidity and mortality. Barton et al. described a case series of 11 patients with HELLP syndrome who underwent needle biopsy of the liver. The pathology was notable for periportal hemorrhage (eight cases), fibrin deposition (six cases), and fatty infiltration (four cases). Only fatty infiltration was demonstrated to be associated with the degree of thrombocytopenia and aminotransferase elevations [21]. In patients with HELLP syndrome, aggressive treatment and prompt delivery should be performed to prevent significant maternal outcomes (morbidity and mortality). Although liver biopsy may be helpful in achieving a diagnosis, it should not delay prompt management in suspected patients.

In cases of severe HELLP syndrome and acute fatty liver of pregnancy, liver biopsy can provide valuable diagnostic information, but it carries significant risks. The main advantage of performing a biopsy is that it allows for a definitive diagnosis, especially when the clinical picture overlaps between HELLP, AFLP, and other causes of liver dysfunction such as viral hepatitis or autoimmune disease. A histologic examination can confirm microvesicular fatty infiltration typical of AFLP or periportal necrosis and fibrin deposition characteristic of HELLP, which can guide further management. However, the disadvantages often outweigh the benefits in these critically ill patients. Because both HELLP and AFLP are associated with thrombocytopenia and coagulopathy, performing a liver biopsy carries a very high risk of bleeding and hepatic rupture, which can be life-threatening. Additionally, the results of the biopsy rarely alter immediate clinical management, as the primary treatment for both conditions is supportive care and prompt delivery. Therefore, in most cases, liver biopsy is avoided [22,23].

Hemophagocytic Lymphohistiocytosis

Yildiz et al. described a case of a 36-year-old woman at 29 weeks of gestation presenting with itchiness and jaundice, lasting a week. She was on medications for an HIV infection three years prior. Her lab results showed acute hepatitis. Further testing was performed to find the origin of the acute hepatitis, including serology, plasma HIV viral load and CD4 count, antinuclear antibodies, rheumatoid factor, and antiphospholipid antibodies. Imaging, including chest x-ray and liver and obstetric ultrasound, was completed. Urinalysis was also performed. All tests were negative. A liver biopsy was performed at 31 weeks and 1 day, and biopsy results were interpreted as either viral or drug-induced hepatitis. The patient developed fever (39°), and therefore, the decision was taken to proceed with an emergency cesarean section at 31 weeks and 6 days of gestation due to the risk of stillbirth. Post-surgery, chorioamnionitis was suspected, and the patient was given ceftriaxone and metronidazole. A week after antibiotics were started, her fever persisted, and antibiotics were stopped. The biopsy was reexamined that led to the diagnosis of hemophagocytic lymphohistiocytosis. A bone marrow biopsy was performed to confirm the diagnosis, and intravenous methylprednisolone was administered for seven days, followed by a tapered dose orally with improvement in the patient’s condition. This case is a significant one, as to the best of our knowledge, it is the first case of HLH proven on liver biopsy in a pregnant patient. To reach this diagnosis, all other liver diseases and obstetric emergencies needed to be ruled out, such as infection, HELLP syndrome, AFLP, etc. Liver biopsy followed by prompt treatment was crucial in the successful recovery of this patient [24].

Intrahepatic Cholestasis of Pregnancy

ICP is a rare obstetric complication (with a prevalence of 0.01%-0.2% in North America and Southern Europe) that commonly presents in the third trimester of pregnancy with pruritus and jaundice. Stulic et al. described the case of a 21-year-old patient admitted during her 11th week of pregnancy, with the first onset of pruritus and jaundice appearing a month before hospitalization. Labs were performed to rule out *Toxoplasma gondii* or viral infection. The patient had deteriorating liver function tests, including aspartate aminotransferase (AST), alanine aminotransferase, alkaline phosphatase (ALP), gamma-glutamyltransferase, and total bilirubin. Ultrasound was then performed and was found to be unremarkable. Finally, the patient underwent a liver biopsy at week 22, and the diagnosis of ICP was made. The patient decided to terminate the pregnancy after consulting with her hepatologist, gynecologist, and anesthesiologist. Her hepatogram returned to normal in a month following the procedure [25]. Thus, in some cases, liver biopsy can assist with confirming the diagnosis of ICP, which can assist in further management.

Liver Mass

**Figure 2:**
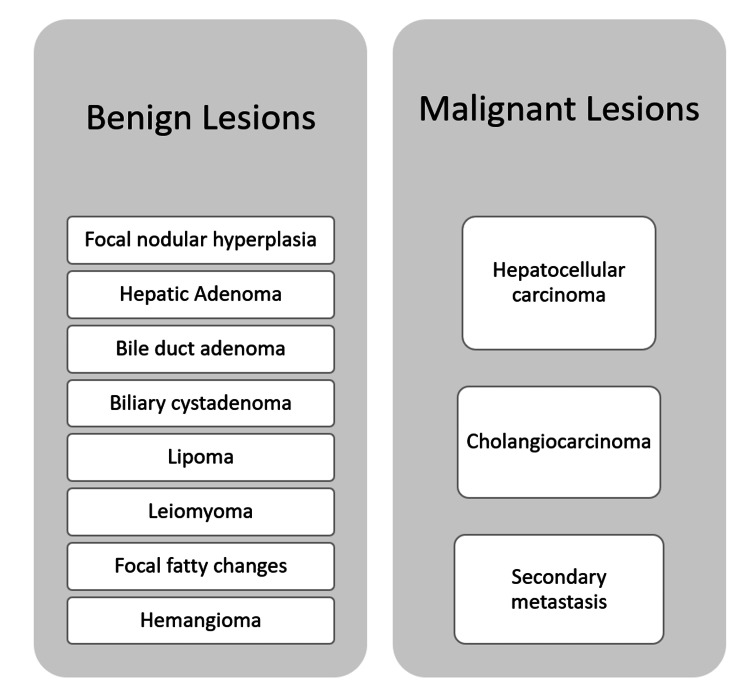
Classification of liver masses Credits: Author's creation

**Figure 3:**
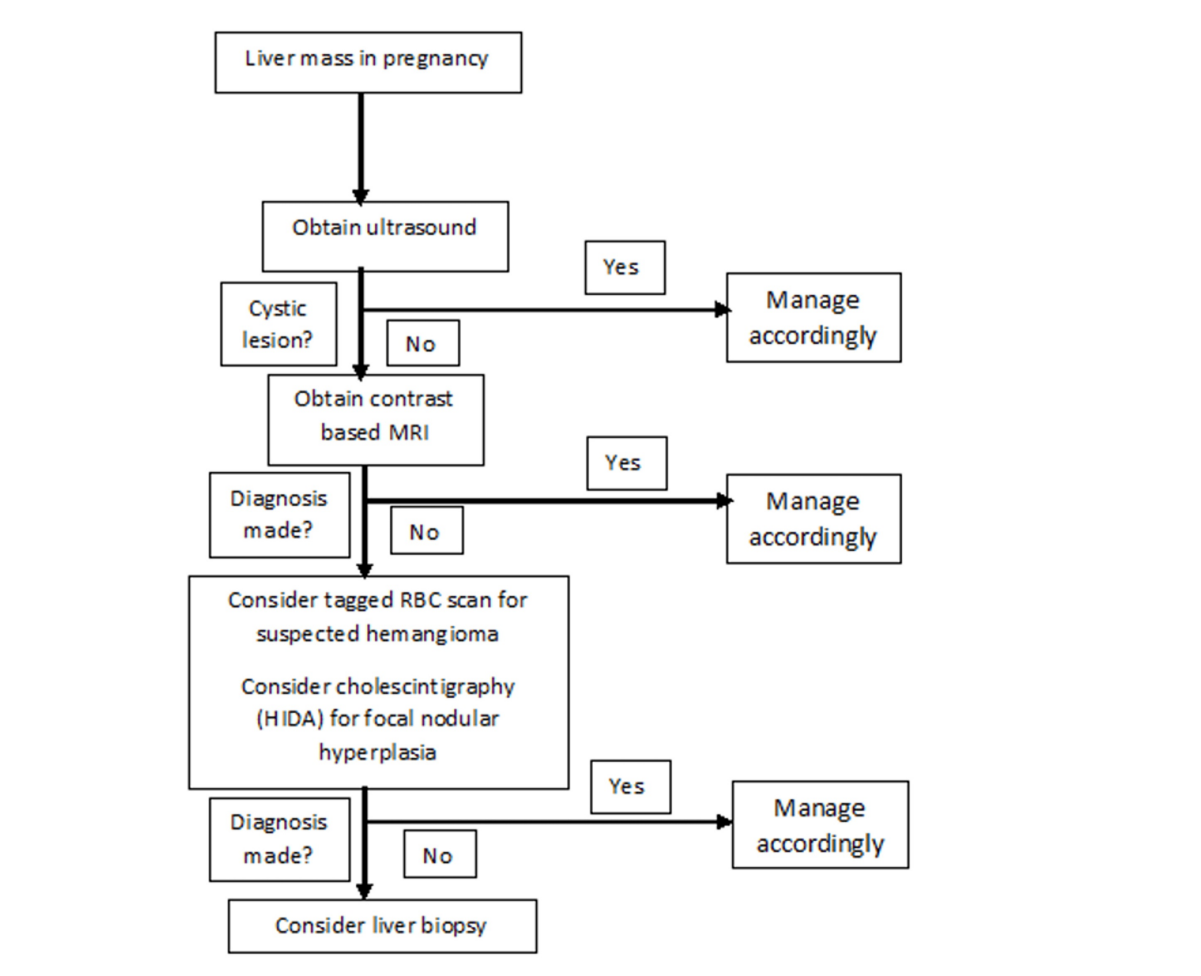
Algorithm for diagnosing a liver mass in pregnancy HIDA: hepatobiliary iminodiacetic acid Credits: Author's creation

Liver masses have a broad differential and can be categorized into benign and malignant masses (Figure 2). A liver biopsy may be necessary for accurate diagnosis; however, this is usually not the first step. Radiological modalities starting with ultrasound, followed by CT/MRI are sufficient in making a diagnosis in many cases. However, in rare cases, a biopsy is needed to confirm the diagnosis (Figure 3).

Hepatic Adenoma

Hepatic adenomas (HAs) are lesions seen in young females and have a strong association with oral contraceptive use. The lesion is also associated with other conditions such as diabetes, steroid use, glycogen storage disease, and iron overload disease state [26,27]. The lesions can be solitary or multiple [27]. Hepatic adenomas are highly vascular tumors that are devoid of a fibrous capsule, thus increasing the risk of hemorrhage if ruptured [27]. The majority of these lesions tend to remain asymptomatic; however, affected patients carry a 27% lifetime risk of hemorrhage, with larger lesions carrying a higher risk [28]. Given the risk of hemorrhage, diagnosis is usually based on radiological assessment by contrast-based CT or MRI. Ultrasonography only highlights the presence of a mass without further details. CT/MRI can reveal hypervascularity and peripheral vessels. In a prospective cohort study of 51 pregnant patients with radiologically and/or biopsy-proven HA <5 cm before pregnancy, only one patient underwent intervention due to the increased size of the lesion (>7 cm) during pregnancy. All other patients were conservatively managed with serial ultrasound examination and had an uneventful pregnancy [29]. In another prospective study of 12 female patients (17 pregnancies), only one patient underwent radiofrequency ablation in the first trimester to treat hormone-sensitive HA. The other pregnancies were managed conservatively [30]. Thus, liver biopsy may not be warranted in these patients due to (1) the availability of an alternative modality for diagnosis, (2) increased risk of hemorrhage, and (3) no significant change in management based on biopsy results.

Hemangioma

Hepatic hemangiomas arise from vascular endothelial cells and are well circumscribed in appearance [31]. Clinically, they are the most common benign lesions present in the liver and rarely present with symptoms [32]. The risk of rupture, necrosis, thrombosis, and hemorrhage is low with these lesions [33]. On ultrasound, well-circumscribed and hyperechoic lesions are noted. A CT scan with contrast will often demonstrate progressive centripetal filling and retained contrast during the delayed phase. Similar filling of lesions and peripheral nodular enhancement is seen with contrast-based MRI [34]. Similar to HA, liver biopsy is not indicated as it would alter management, and radiological assessment is sufficient for making a diagnosis.

Focal Nodular Hyperplasia

Focal nodular hyperplasia is the second most common benign tumor of the liver characterized by the presence of a well-circumscribed lesion with a central stellate scar extending peripherally [32]. On ultrasound, the lesion can appear as hypoechoic or isoechoic and may have a central scar. CT and MRI can reveal a central scar and a mass surrounded by a pseudocapsule [34]. These lesions are mostly asymptomatic, and the risk of rupture is extremely low [35]. Serial radiological assessments are appropriate with no particular indication for biopsy.

Hepatocellular Carcinoma

Hepatocellular carcinoma (HCC) is usually rare in pregnancy and is extremely aggressive [36]. The condition is less common in female patients, and the fact that HCC usually arises in the setting of cirrhosis, which is often associated with infertility, further decreases its prevalence in pregnant patients [37]. Estrogen is thought to accelerate the progression of HCC, as shown in a comprehensive review of 33 cases by Cobey and Salem. Of 33 cases, 20 patients died within a few days of presentation [31]. Although liver biopsy is associated with hemorrhage and possible tumor seeding, it may still be warranted in cases with large tumors (>5 cm) and exclusion of other possible masses on radiological assessment [31]. To our knowledge, only one case has been reported in the literature where the diagnosis was made through obtaining a liver biopsy during pregnancy. The patient was a 28-year-old woman who was found to have an elevated alpha-fetoprotein level (8400 ng/ml) in her 18th week of pregnancy. Her other labs included a total bilirubin level of 8 µmol/l, an ALP level of 228 IU/l, and an AST level of 78 IU/l. Ultrasound showed an enlarged liver with multiple lesions in the liver. The liver biopsy was consistent with HCC in a non-cirrhotic liver. The decision was made to terminate the pregnancy, and chemotherapy was initiated. The patient eventually died 12 months after presentation [37].

Liver Transplant

Given the increasing number of liver transplants performed each year, there is also a simultaneous increase in the number of female patients receiving a liver transplant in their reproductive years and then subsequently getting pregnant shortly after the transplant. A single-center experience described by Christopher et al. reported 12 cases of pregnant women undergoing ultrasound-guided liver biopsy who were found to have acute cellular rejection. No further complications were observed, and the rejection was appropriately managed [38]. Another study reported two cases of liver biopsy during pregnancy (one at 10 weeks and another at 35 weeks of gestation). The authors did not comment regarding adverse outcomes with respect to the biopsy procedure, but did report that biopsies showed acute graft rejection [39]. Thus, liver biopsy is likely warranted in pregnant women who have undergone liver transplantation if there is a suspicion of graft rejection.

Outcomes of liver biopsy with respect to indications

In cases of AFLP, liver biopsy is not usually performed due to the effectiveness of the Swansea score in predicting AFLP, avoiding any invasive diagnostics during pregnancy. In the case report of Onwuagbu et al., the fetus was delivered at 25 weeks, 3 days of gestation, and died four days postpartum due to prematurity [20]. Due to the authors citing prematurity as the reason for fetal death, we are unable to conclude if biopsy had any effect on this outcome.

In Yildiz’s case report of hemophagocytic lymphohistiocytosis, the authors were unable to confirm a diagnosis without liver biopsy and bone marrow biopsy. No information on maternal or fetal outcomes, other than the patient completely recovering and not having any relapse after four months of follow-up, was provided [24]. This is a rare case where a biopsy was needed to obtain a diagnosis, rather than relying on noninvasive testing.

Liver biopsy was crucial in diagnosing ICP in the case described by Stulic et al. The dangerous risks to the fetus, of ICP, during the first trimester were presented to the patient, and she decided to terminate the pregnancy [25].

Lastly, in a case described by Lau et al., a pregnant woman underwent biopsy in her 18th week of pregnancy, which was crucial as it led to the diagnosis of HCC. The patient eventually terminated the pregnancy to further manage her HCC [37].

Adverse effects of liver biopsy on pregnancy

Currently, the most comprehensive study of adverse outcomes of liver biopsy in pregnancy has been conducted by Ludvigsson et al. The authors conducted a nationwide cohort study in Sweden and used data from the Swedish Medical Birth Registry from 1992 to 2011. The investigators hypothesized that liver biopsy may have adverse pregnancy outcomes, with the two main outcomes being preterm birth and stillbirth. It was found that in the 1,960,402 births in 1,135,983 women in Sweden during 1992-2011, only 23 liver biopsies were performed. The authors compared three groups: pregnancies with liver biopsy, pregnancies with prior liver disease but no biopsy, and the general population. Seventeen of the 23 women had a liver diagnosis before pregnancy. Indications to warrant biopsy included hepatitis C (7), chronic hepatitis (3), hepatitis B (1), alcoholic hepatitis (1), autoimmune hepatitis (2), abnormal serum enzymes (2), connective tissue disease (1), malignancy (including metastasis) (3), Crohn’s disease (1), and liver fibrosis (1); one was not documented. In the 23 pregnancies with liver biopsies, there were no stillbirths, compared to stillbirths in 0.3% of the general population. We predict that this could be due to the small sample size of pregnancies in which a liver biopsy was done. Preterm birth, however, was found to be more common, with women giving birth an average of 10 days earlier than the general population, and 6 days earlier than women with liver disease, but with no instance of liver biopsy. Three of 23 (13%) neonates were found to be SGA compared to 3% in the general population. The average weight difference was -367 g compared to the general population, and -391 g compared to women with liver disease but no biopsy [3].

Of the 23 biopsies, 13 were performed during the first trimester. The authors found this to be a limitation and did not have enough data on second- or third-trimester liver biopsies. Other limitations noted were that women who underwent liver biopsy were inherently less healthy and more prone to birth complications. The type of sedation used during biopsy was not recorded, which could have also played a role in birth outcomes. None of the pregnancies included women with AFLP, a rare, but possibly fatal disease [3].

Heneghan and Cannon, in an article with the goal of looking at the management of liver disease in pregnancy, examined the study by Ludvigsson et al. and commented that many of the first-trimester biopsies were performed early, likely due to the women not even knowing that they were pregnant. This could also have led providers to decide to proceed with a liver biopsy with less hesitation. In addition to reviewing the Swedish cohort study, they also provided a workup helpful when managing liver disease during pregnancy. This begins with examining liver enzymes and proceeding to ultrasound. If a mass is found, then a biopsy is recommended if the mass is suspected to be malignant. Again, it is noted that there is a limited amount of data on liver biopsy during pregnancy, possibly due to the introduction of the Swansea criteria in 1999, which is now the gold standard for diagnosing AFLP. If a certain number of criteria are met, then there is no need for a liver biopsy [40].

## Conclusions

After reviewing case reports, case series, and other studies, we conclude that liver biopsy is a useful, but not always necessary, diagnostic tool for the evaluation of liver disease during pregnancy. Although the data is limited on the number of liver biopsies performed during pregnancy, few adverse maternal or fetal outcomes have been documented. When liver disease of unknown etiology is present, biopsy remains the most useful procedure to aid diagnosis when all other measures (labs, ultrasound, etc.) have been taken. Our findings remain in agreement with the current guidelines for the management of liver disease in pregnancy as established by the American College of Gastroenterology and American Association for the Study of Liver Diseases. As long as there is a logical flow of events (least invasive to most invasive), we see no reason to exclude the use of liver biopsy during pregnancy. Future studies are needed to further address the utility and safety of liver biopsy during pregnancy.
